# Nepalese Medical Students in the COVID-19 Pandemic: Ways Forward

**DOI:** 10.31729/jnma.4924

**Published:** 2020-05

**Authors:** Mandeep Guragai

**Affiliations:** 1 Kathmandu Medical College and Teaching Hospital, Sinamangal, Kathmandu, Nepal.

**Keywords:** *COVID-19*, *medical students*, *outbreak*, *pandemic*

## Abstract

The coronavirus disease 2019 has not hit Nepal as hard as it has the rest of the world (as of 4th April 2020). Countries are reporting a saturation in healthcare facilities and facing a rise in demand for human resources for health. It is difficult to predict the extent of the disease transmission in Nepal in the absence of epidemiologic and statistical analysis in our context. But based on calculations made by epidemiologists in other countries, there seems to be a significant possibility of an outbreak in our communities too. Medical students can be a valuable human resource in a variety of ways to aid in the country's response to a possible outbreak. However, their involvement in the pandemic comes with its own challenges. Thorough planning and preparation must be done before allowing medical students to take part in the battle against the pandemic.

## INTRODUCTION

The World Health Organization (WHO) reports that as of April 11, 2020, the coronavirus disease 2019 (COVID-19) has spread throughout the world with 1,610,909 confirmed cases and 99,690 mortalities globally.1 As of April 11, 2020, there are 15 patients in isolation in different hospitals, and 9 confirmed cases of COVID-19 out of 4426 tests done in Nepal.^2^The disease is highly contagious and no proven treatment or vaccine for prevention is available yet. In such a context, the future will present substantial challenges.

Countries that have been affected the most have reported a significant scarcity of health resources resulting in a depleted and overworked human resource for health (HRH). From a public health perspective, mitigation measures (like social-distancing) that have been put into place in Nepal aim to flatten the epidemic curve so that those who are infected can be managed without saturating available health resources. Though we can hope for a future where the number of COVID-19 positive cases that require health services at any given time remains in proportion with the available resources, globally a much scarier picture is being seen. COVID-19 had a doubling time of 4-5 days, in its early stages in China.^3^ Although this data is from the time when a lockdown was not put into place in China, it shows how easily the spread could go out of hand.

Preparations must factor in the possibility of a lack of human resources during an outbreak. Utilizing doctors, nurses, paramedics, and all auxiliary healthcare providers might not be enough if such a situation arises. With an eminent risk of the frontline workers themselves being infected and having to be isolated until recovery present, it would be unwise to falter in deciding how to maintain an adequate human resource. Thus, the country might have to deploy medical students in response. As a matter of fact, the Ministry of Health and Population (MoHP) has asked state governments to deploy medical students studying with a government scholarship if necessary.

## GLOBAL SCENARIO

- Several governments have alluded the possible use of medical students in health systems affected by COVID-19.^4^
- There are medical schools that are allowing students to graduate early or return to the clinical environment to help address impending staff shortages.^5^
- A group of Harvard medical students have prepared a faculty reviewed COVID-19 curriculum.^6^
- Hundreds of medical students in the United States (US) have contributed to collect and distribute surgical and N95 masks, deployed to call-centers, provided child-care services and used technology and innovation to help in different ways.^7^ Similar initiatives have been taken in other countries too.

## WHAT CAN MEDICAL STUDENTS DO

Medical students can take inspiration from peers in other countries. Simply, medical students can help spread authentic information on COVID-19 and aid in risk communication and community engagement (RCCE). They can explain their families and community members the relevance of social distancing and handwashing practices, give them knowledge on mitigation and containment and their effectiveness in controlling the disease spread, and explain the ways in which the disease transmits and the methods of preventing it. Teaching people the proper ways of handwashing could contribute in putting a stop to the transmission. Many people might be having seasonal flu, whose symptoms are like those exhibited by patients with mild cases of COVID-19, causing unnecessary stress and anxiety in them. Although the diagnosis should be made by a licensed practitioner, a medical student, based on sound scientific evidence can counsel them about the prognosis and possible outcomes, and warn them about the danger signs following which they should ought for medical care immediately.

Creative students can help spread credible information via social media and digital platforms through infographics made in an understandable language. A further step would be to translate such information in a local language, which can increase the reach of the information and engage more people. For example, I have collaborated with the COVID-19 student response team of the Harvard Medical School to translate graphical summaries of their COVID-19 curriculum into Nepali.^6^

On a larger scale, the government can deploy medical students to trace undiagnosed cases of COVID-19. They can assist the case investigation and contact tracing teams in screening large groups of people for the disease via rapid diagnostic tests or in sample collection for the polymerase chain reaction test. This could be valuable training for medical students; which they wouldn’t get through the usual curriculum. They can also assist in distributing humanitarian aid, like food and necessities to the needy.

Without the risk of an exposure, medical students can be placed in call-centers to offer guidance to concerned individuals about their symptoms, and provide information on infection control measures, when to visit a doctor, where to get tested, what hospitals have been assigned to treat COVID-19 cases, etc.

## CHALLENGES

Although deploying medical students alongside physicians seems like a great idea, it does come with its challenges. Firstly, the ethical issue of whether or not to expose unlicensed students to the direct risks of transmission has to be given much thought. Due to a limited experience, putting the safety of students, other healthcare providers and patients in danger by deploying them would depend on the extent and severity of the outbreak and the demand of the situation. Training medical students before deployment would be one way to come around this challenge. However, in case of an already stretched-out and overworked human resource during the outbreak, it would be difficult to organize such training.

Guidelines and standard operating procedures (SOP) for medical students must be prepared beforehand to provide a clear description of what roles are to be played. Since, students are not licensed practitioners, they are not bound by the same governing legal and ethical provisions as are health professionals. For example, there are ethical codes, guidelines, Nepal Medical Council (NMC) act and regulation for medical practitioners. However, the same could also not be used to govern medical students. So, a description of their roles and responsibilities, ethical and legal guidelines, and terms of reference describing what to do and what not to do when faced with a difficult situation must be provided to students.

Putting students in the frontline would mean that they are exposed not just to the infection, but also to the immense physical and mental stress the situation brings. Watching patients in distress, and debilitated patients lose their lives might leave a severe psychological impact on them.

Medical students are a trained cohort of people with knowledge of the disease, epidemiology, and principles of infection transmission and control. Technical information and concepts can be simpler to grasp for the students in comparison to others. Thus, it would be effective, time-efficient, and would require fewer efforts to train them for certain roles in the battle against the outbreak. However, it would require thorough planning and proper guidelines and a collaboration with multiple sectors before bringing it into practice.

## Conflicts of Interest:

**None.**
